# Extracorporeal shockwave relieves endothelial injury and dysfunction in steroid-induced osteonecrosis of the femoral head via miR-135b targeting FOXO1: *in vitro* and *in vivo* studies

**DOI:** 10.18632/aging.203816

**Published:** 2022-01-07

**Authors:** Xinjie Wu, Yanlei Wang, Xiaoyu Fan, Xin Xu, Wei Sun

**Affiliations:** 1 Peking University China-Japan Friendship School of Clinical Medicine, Beijing 100029, China; 2 Department of Orthopedic Surgery, China-Japan Friendship Hospital, Beijing 100029, China; 3 Beijing University of Chinese Medicine, Beijing 100029, China; 4 Graduate School of Peking Union Medical College, Beijing 100730, China

**Keywords:** shockwave, osteonecrosis, microRNA, FOXO1

## Abstract

Injury and dysfunction of endothelial cells (ECs) are closely related to the pathogenesis of steroid-induced osteonecrosis of the femoral head (ONFH), while MicroRNAs (miRNAs) play an essential role in the processes. Extracorporeal shockwave treatment (ESWT) has been used in the non-invasive treatment of various diseases including musculoskeletal and vascular disorders. In particular, ESWT with low energy levels showed a beneficial effect in ischemic tissues. However, there has been no comprehensive assessment of the effect of ESWT and miRNAs on steroid-induced ONFH. In the present study, we investigated the role and mechanism of ESWT and miRNAs both *in vitro* and *in vivo*. Using a steroid-induced ONFH rat model, we found that ESWT significantly enhances proliferation and angiogenesis as well as alleviates apoptosis. In two types of ECs, ESWT can promote cell proliferation and migration, enhance angiogenesis, and inhibit apoptosis. Notably, our study demonstrates that miR-135b is downregulated and modulated forkhead box protein O1 (FOXO1) in ECs treated with dexamethasone. Remarkably, both miR-135b knockdown and FOXO1 overexpression reversed the beneficial effect of ESWT on ECs. Additionally, our data suggest that ESWT activates the FOXO1-related pathway to impact proliferation, apoptosis, and angiogenesis. Taken together, this study indicates that ESWT relieves endothelial injury and dysfunction in steroid-induced ONFH via miR-135b targeting FOXO1.

## INTRODUCTION

Osteonecrosis is regarded as bone cell death initiated by an impairment of the blood supply to the bone [1]. Steroid-induced osteonecrosis of the femoral head (ONFH) is a serious adverse result of excessive usage of glucocorticoids (GCs), which is one of main causes of ONFH [2]. While the exact pathogenesis and molecular mechanisms that contribute to ONFH remain indefinite, it seems that endothelial cells (ECs) injury and dysfunction play a crucial role in the course of the disease.

In the past, microRNAs (miRNAs/miRs) are deemed as “noise” or “junk” RNAs. However, they are now broadly recognized a critical regulator of gene expression that function mainly at the posttranscriptional level. The progress in high-throughput sequencing techniques and associated analyses have shed light on the characters of miRNAs in ONFH development and progression [3]. Recently, extracorporeal shockwave treatment (ESWT) has been proven to be able to enhance angiogenesis via stimulating localized stress on the membranes of ECs, which is similar to fluid shear stress in blood vessels [4, 5]. However, a comprehensive assessment of the effects of ESWT and miRNAs on steroid-induced ONFH has still not been reported. In this study, we explored the potential role of ESWT in the proliferation, apoptosis, migration, and angiogenesis of ECs. In addition, we attempted to obtain insight into the molecular and signaling mechanisms of effects of ESWT on ONFH.

## MATERIALS AND METHODS

### Ethics statement

The study was approved by the Institutional Ethics Review Committee of the China-Japan Friendship Hospital (No. 2016-GZR-4, Beijing, China). All animal experiments were performed following the recommendations of the Guide for the Care and Use of Laboratory Animals, published by the National Institutes of Health (NIH). The approved guidelines and regulations were followed in all experiments, and informed consent was obtained from all study patients.

### Animal grouping and establishment of the ONFH rat model

The Animal Research Committee of the China-Japan Friendship Hospital approved all procedures carried out. A total of 48 male Sprague-Dawley rats aged 12 weeks and weighing 300 ± 20 g were obtained from the Laboratory Animal Center of the Academy of Military Medical Sciences (animal license no. SCXK (Jun) 2017-0004; Beijing, China). They were randomly assigned to the following groups: (a) the control group (n = 12), (b) the ESWT group (n = 12), (c) the methylprednisolone (MP) group (n = 12), and (d) the MP + ESWT group (n = 12). Rats in the model group were administrated 20 μg/kg lipopolysaccharide (LPS; Sigma, San Francisco, CA, USA) via intraperitoneal injection. Then, 24 h later, the rats got three doses of intramuscular injections of 40 mg/kg methylprednisolone (Pfizer Inc., Ascoli Piceno, Italy) at intervals of 24 hours as previously described [6]. Rats in the control group were administrated the same volume of normal saline.

### ESWT

After shaving both hindlimbs, the rats were placed on a warming pad for ESWT. Based on a previous study [7], the shock wave applicator (Dornier AR2; Wessling, Germany) with a probe was placed on the hip, and a single bout of 1,500 shocks was delivered at an energy density of 0.5 mJ/mm^2^ and a frequency of 1 Hz for 25 min. For cells underwent ESWT, 1 × 10^6^ cells were harvested and resuspended in 1 ml of culture medium in microcentrifuge tubes. The shockwave applicator was maintained just on the surface of tubes to treat cells via gels.

### Angiography and micro-computed tomography (CT) scanning

Six weeks after the final MP injection, angiography and micro-CT scanning were performed to analyze the microstructure of the femoral heads. After general anesthesia via the administration of phenobarbital sodium, 4% paraformaldehyde were perfused into the aorta ventralis of the open abdominal cavity followed by MICROFIL (MV-112; Flow Tech, Inc., Carver, MA, USA) [8]. Subsequently, the bilateral femoral heads were harvested and stored at 4° C overnight. After decalcification, all samples were scanned with a micro-CT imaging system (Quantum GX; PerkinElmer, Waltham, MA, USA), and the total vessel volume was quantified with Analyze 12.0 software (PerkinElmer).

### Immunohistology

Six weeks after the final MP injection, all rats were sacrificed, and the femurs were fixed with 4% paraformaldehyde for 24 hours at 4° C. Then the samples underwent decalcification with 10% ethylenediaminetetraacetic acid (pH 7.4) for 8 weeks. After that, they were dehydrated in different concentrations of ethanol, embedded in paraffin, and cut into 5-μm-thick sections. Assessment was carried out with a light microscope.

For ECs analysis, anti-CD31 antibody (Abcam, Cambridge, UK), anti-Ki-67 antibody (Bioworld, Nanjing, China), and terminal deoxynucleotidyl transferase dUTP nick end labeling (Beyotime, Shanghai, China) were applied to stain the samples according to our previous study. ImageJ software (NIH, Bethesda, MD, USA) was used to analyze the immunohistochemical staining images in terms of integrated option density (IOD) of the total area of trabecular bones and the target protein. Then, the mean density (IOD/area) was assessed.

### Cell culture and treatment

Using the previously described protocols [9], human umbilical vein ECs (HUVECs) purchased from the Cell Bank of Shanghai Institute of Biological Science (Shanghai, China) were cultured. HUVECs from passages 3 to 5 were used in this study. Primary human bone microvascular ECs (BMECs) were isolated from patients with femoral neck fractures who were undergoing hip arthroplasty (Supplementary Table 1). BMECs were cultured using a previously described protocol [10]. BMECs from passages 3 to 5 were utilized in all experiments. The expression levels of the ECs markers CD31 (Proteintech, Rosemont, IL, USA) and vWF (Proteintech) were examined using immunofluorescence. To explore the effects of ESWT, the cells were either treated with dexamethasone (DEX; Yuanye, Shanghai, China) or underwent ESWT or both. At certain intervals, a series of *in vitro* assays were carried out on the cells.

### Human femoral head tissues

As described previously, surgery-isolated femoral head tissues from patients with steroid-induced ONFH and paired tissues from patients with femoral neck fractures were collected from five patients.

### Cell viability assay

The Cell Counting Kit-8 assay (CCK-8; Dojindo, Kumamoto, Japan) was utilized to estimate cell viability. At 24h, 48h, and 72 h, 10 μL of CCK-8 solution was added to the cells in each well and incubated at 37° C for 1 h. The absorbance was evaluated at 450 nm using a microplate reader.

### Cell proliferation assay

To confirm the changes in the proliferation rates of the two cell types (HUVECs and BMECs), an EdU-488 Cell Proliferation Kit (Ribobio, Guangzhou, China) was used, and the results were assessed via fluorescence microscopy or flow cytometry (FC) per the manufacturer’s instructions.

### Apoptosis assay

Annexin-V-fluorescein isothiocyanate (FITC) and propidium iodide (PI) assays (Beyotime) were used to measure cell apoptosis. The cells were incubated with Annexin V-FITC and PI reagent for 15 min in the dark. Then, the cell apoptosis rate was evaluated using FC.

### Migration assay

To observe the effects of DEX and/or ESWT on the migration of the two cell types (HUVECs and BMECs), wound healing and transwell assays were utilized. A scratch was created on a cell monolayer by using a 200 μL pipette tip. At 0 h and 24 h, the width of the scratch was assessed, and the rate of scratch recovery or the number of migrated cells was determined. For the transwell assays (3 μm; Corning, Corning, NY, USA), 1 × 10^5^ cells/well treated with different factors were suspended in the upper chamber of a 24-well transwell plate with a 200 μL serum-free medium. Next, 600 μL/well complete medium with 20% serum was added in the lower chamber. After twelve hours, the insert was stained with 0.1% crystal violet. Subsequently, the cells attached to the upper surface of the filter membranes were cleared with a cotton swab. Migration activity was assessed under an optical microscope.

### Tube formation assay

To explore the vessel-like construction activity of HUVECs and BMECs, pre-cooled 24-well plates were coated with Matrigel (289 μL) at 37° C for 30 min. The two cell types were seeded (4.0 × 10^5^ cells/mL) onto the Matrigel-coated plates at 37° C and 5% CO_2_ for 16 h. Images were acquired using an optical microscope, and the results were analyzed by using ImageJ software (NIH).

### Cell transfection

The miR-135b inhibitor, forkhead box protein O1 (FOXO1) overexpression vector, and negative control were obtained from Hanbio (Shanghai, China). Lipofectamine 3000 (Invitrogen, Carlsbad, CA, USA) was used to introduce miRNA inhibitors into HUVECs or BMECs per the manufacturer’s protocol.

### Luciferase reporter assays

Luciferase reporters containing wild-type or mutated plasmid were constructed using psi-CHECK2 vectors (Promega, Madison, WI, USA). The luciferase vector containing the pcDNA3.1-FOXO1-3'-untranslated region plasmid was co-transfected with the miR-135b mimic or negative control into human embryonic kidney 293 (HEK293) cells using Lipofectamine 3000 (Invitrogen), with 10 ng of Renilla luciferase reporters used as an internal control. After 48 h, the cells were harvested and lysed. Luciferase activity was determined using the Dual-Luciferase Reporter Assay System (Promega) per manufacturer’s instructions.

### RNA extraction and quantitative reverse transcription polymerase chain reaction (qRT-PCR)

Total RNA was obtained using TRIzol reagent (Invitrogen) and reverse transcribed using a miRNA reverse transcription kit (Cat. no. 4366596l; Applied Biosystems, Foster City, CA, USA). The expression of miR-135b was quantified using TaqMan Universal Master Mix II (Cat. no. 4440038; Applied Biosystems). The primes for miR-135b were TaqMan Assays and U6 served as an internal control for this assay (Cat. no. 4426961; Applied Biosystems). The 2^-ΔΔCT^ method was used to measure the relative levels.

### Western blot analysis

HUVECs and BMECs were lysed using radioimmunoprecipitation assay buffer (Biosharp, Hefei, China). To determine protein concentration, a Bicinchoninic Acid Protein Assay Kit (Biosharp) was utilized. Equivalent protein lysates were separated using sodium dodecyl sulfate polyacrylamide gel electrophoresis and transferred onto polyvinylidene difluoride membranes. Then they were then subjected to western blot analysis. The primary antibodies against FOXO1 (2880S), cleaved caspase-3 (9661S), and β-actin (3700S) were purchased from Cell Signaling Technology (Danvers, MA, USA). The primary antibodies anti-cyclin D1 (60186-1-1), anti-cyclin E1 (11554-1-AP), anti-Bim (22037-1-AP), anti-Bcl2 (60178-1-Ig), anti-Bax (60267-1-Ig), anti-matrix metalloproteinase (MMP)2 (66366-1-Ig), anti-MMP9 (N-terminal, 10375-2-AP), and anti-vascular endothelial growth factor A (VEGFA; 66828-1-Ig) were obtained from Proteintech. In addition, horseradish peroxidase-conjugated goat anti-mouse or anti-rabbit IgG H&L (ZJ2020-M and ZJ2020-R, respectively; Bioworld) was applied as the secondary antibody. The protein bands were visualized via an electrochemiluminescence detection system (Thermo Fisher Scientific, Waltham, MA, USA) and analyzed using Image Lab 5.0 software (Bio-Rad, Hercules, CA, USA).

### Bioinformatics analysis

The three most popular databases, namely TargetScan (http://www.targetscan.org/vert_72/), miRDB (http://www.mirdb.org/), and miRWalk (http://mirwalk.umm.uni-heidelberg.de/), were used to predict the target genes of miR-135b. Only those identified in all three databases were determined as target genes of miR-135b. ClueGO, a Cytoscape plug-in, was used to integrate Gene Ontology (GO) terms and Kyoto Encyclopedia of Genes and Genomes (KEGG) pathways and create a functionally organized GO term/KEGG pathway network [11].

### Statistical analysis

All statistical analyses were performed using GraphPad Prism software (version 8.0; GraphPad Software Inc., La Jolla, CA, USA). All values are expressed as means ± standard deviations. Statistically significant differences were evaluated using the Student’s *t*-test or a one-way analysis of variance. Data with a P value < 0.05 was considered to be significant.

### Availability of data and material

The data of this study are available from the corresponding author on reasonable request.

## RESULTS

### ESWT enhances proliferation and angiogenesis and alleviates apoptosis in steroid-induced ONFH in rats

**Figure 1:**
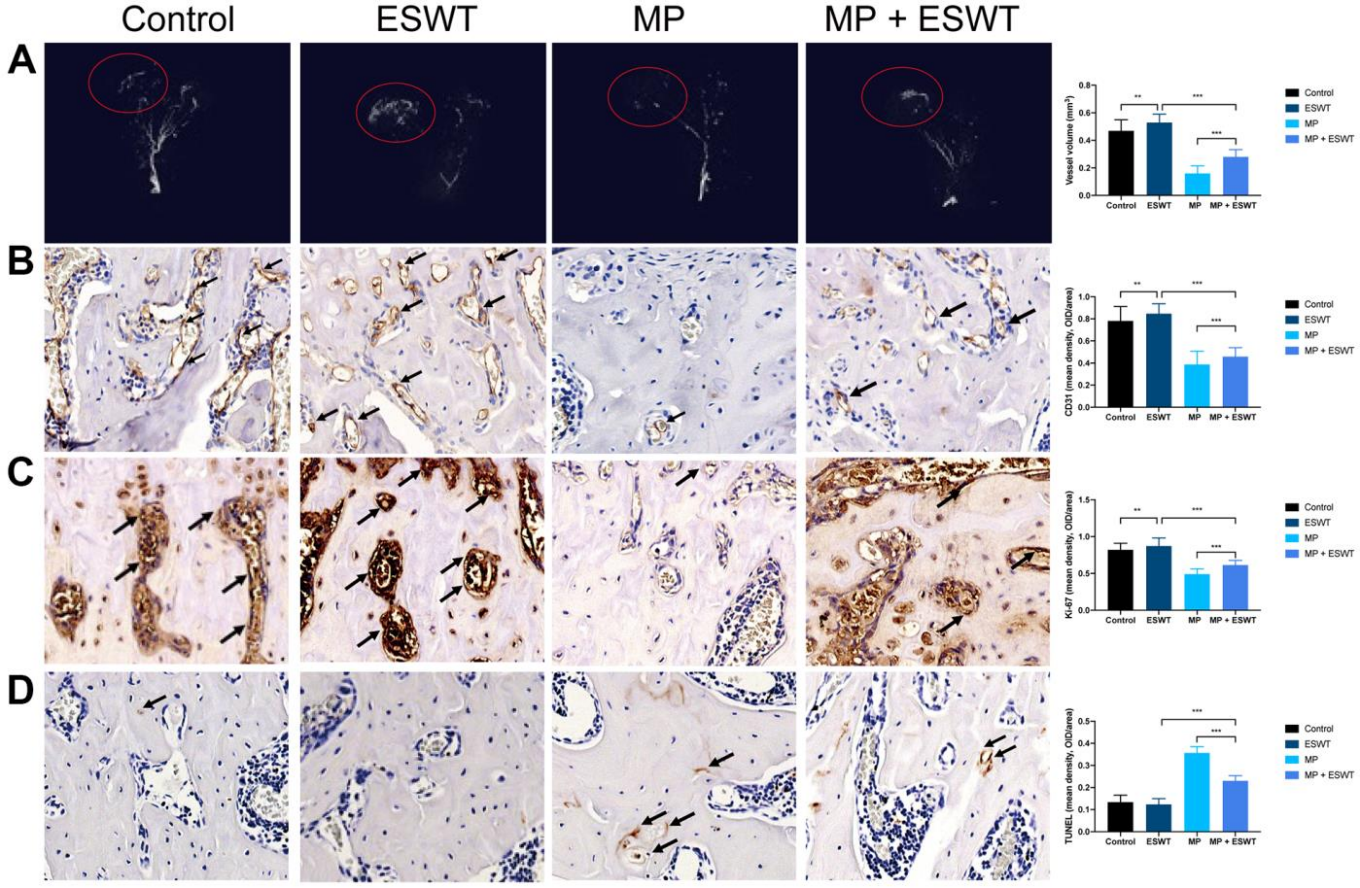
**Effect of ESWT on proliferation, angiogenesis, and apoptosis in steroid-induced ONFH in rats.** (**A**) blood supply assessed by micro-CT scanning; (**B**) CD31 staining in the femoral head; (**C**) Ki-67 staining in the femoral head; (**D**) TUNEL staining in the femoral head. (magnification, ×20) n=6 ^**^P < .01, ^***^P < .001.

Here, we evaluated the damage to the vessels in the femoral head. Angiography, micro-CT scanning, and ECs analysis suggested that MP obviously destroyed the vessels of the femoral head. However, ESWT resulted in significant angiogenesis in the femoral head (Figure 1A, 1B).

The effects of ESWT on cellular proliferation in the femoral heads of animal models were assessing using Ki67 immunostaining. The results revealed that cellular proliferation in the MP group was reduced compared to that in the control group. However, ESWT significantly reversed the steroid-induced anti-proliferative effect (Figure 1C). As shown in Figure 1D, the apoptosis rate was higher in the MP group than in the control group. Conversely, ESWT alleviated apoptosis in the femoral head. The aforementioned results suggest that ESWT enhances ECs proliferation and angiogenesis as well as alleviates apoptosis in steroid-induced ONFH.

### Identification of BMECs

The isolated cells showed high expression levels of CD31 and vWF (Supplementary Figure 1). These results demonstrated that the cells were BMECs, which were utilized in the experiments described below.

### Effect of ESWT on the proliferation of ECs treated with glucocorticoids (GCs)

**Figure 2:**
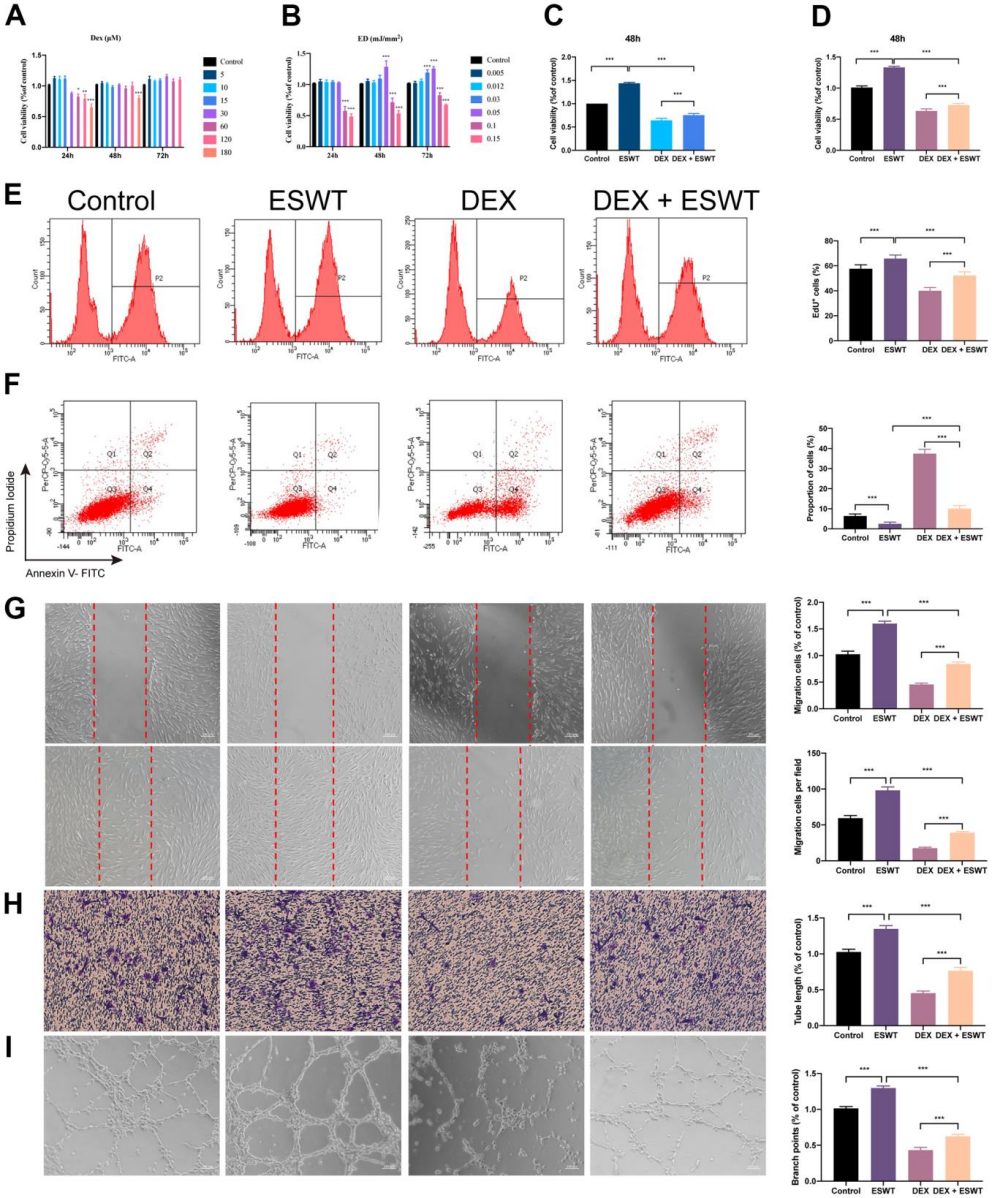
**Effect of ESWT on endothelial cells treated with GCs.** (**A**–**C**) cell viability was examined by CCK-8 analysis in HUVECs; ECs were subjected to ESWT with 0.05 mJ/mm^2^, 1000 shots followed by DEX with 180 μM. (**D**) cell viability examined by CCK-8 analysis in BMECs; (**E**) cell proliferation confirmed by EdU assay in BMECs; ECs were subjected to ESWT with 0.03 mJ/mm^2^, 1000 shots followed by DEX with 180 μM. (**F**) apoptosis rate of assessed through Annexin V-FITC/PI in BMECs; ECs were subjected to ESWT with 0.05 mJ/mm^2^, 1000 shots followed by DEX with 180 μM. (**G**) migration ability evaluated by wound healing assay in BMECs; (**H**) migration ability evaluated by Transwell assay in BMECs; (**I**) angiogenesis ability evaluated by tube formation assay in BMECs. n=3 ^**^P < .01, ^***^P < .001.

To explore the functional roles of ESWT on the proliferation of ECs treated with normal or high levels of GCs, HUVECs and BMECs were cultured in a conditioned medium with DEX and/or ESWT for a series of functional assays. Cell proliferation was observed using the CCK-8 assay (Figure 2A–2D) and confirmed using the EdU assay (Figure 2E). The results showed that DEX markedly reduced the proliferative capability of the cells, whereas ESWT led to a significant increase in the proliferation of the two cell types. In addition, the DEX-induced downregulation of cell proliferation could be ameliorated by ESWT in the two cell types (Supplementary Figure 2).

### Effect of ESWT on the apoptosis of ECs treated with GCs

We used Annexin V-FITC/PI double staining with FC analysis to assess cell apoptosis. The results demonstrated that DEX dramatically increased the apoptosis rate in cells compared to that in the control group. Conversely, pretreatment with ESWT attenuated the apoptotic effect of DEX on HUVECs and BMECs. In addition, HUVECs and BMECs exposed to ESWT alone did not exhibit more apoptotic cells compared to those in the control group (Figure 2F and Supplementary Figure 2).

### Effect of ESWT on the migration and angiogenesis of ECs treated with GCs

To determine whether ESWT affects the migration and angiogenesis of ECs with DEX, we performed wound healing, transwell, and tube formation assays. The results of migration *in vitro* showed that DEX imped the wound recovery and migration of ECs, whereas ESWT ameliorate these processes. Furthermore, we found that HUVECs and BMECs treated with ESWT alone demonstrated higher migration and wound recovery rates compared to the control group (Figure 2G, 2H and Supplementary Figure 2).

The DEX group showed strong anti-angiogenic effects in the tube formation assay with smaller tube length and loop formation, which was ameliorated by ESWT treatment. Furthermore, we observed that both types of ECs treated with ESWT alone presented superior angiogenesis activity compared to that of the control group (Figure 2I and Supplementary Figure 2).

### miR-135b is downregulated in DEX-treated human ECs and displays decreased levels in human necrotic femoral head tissues

**Figure 3:**
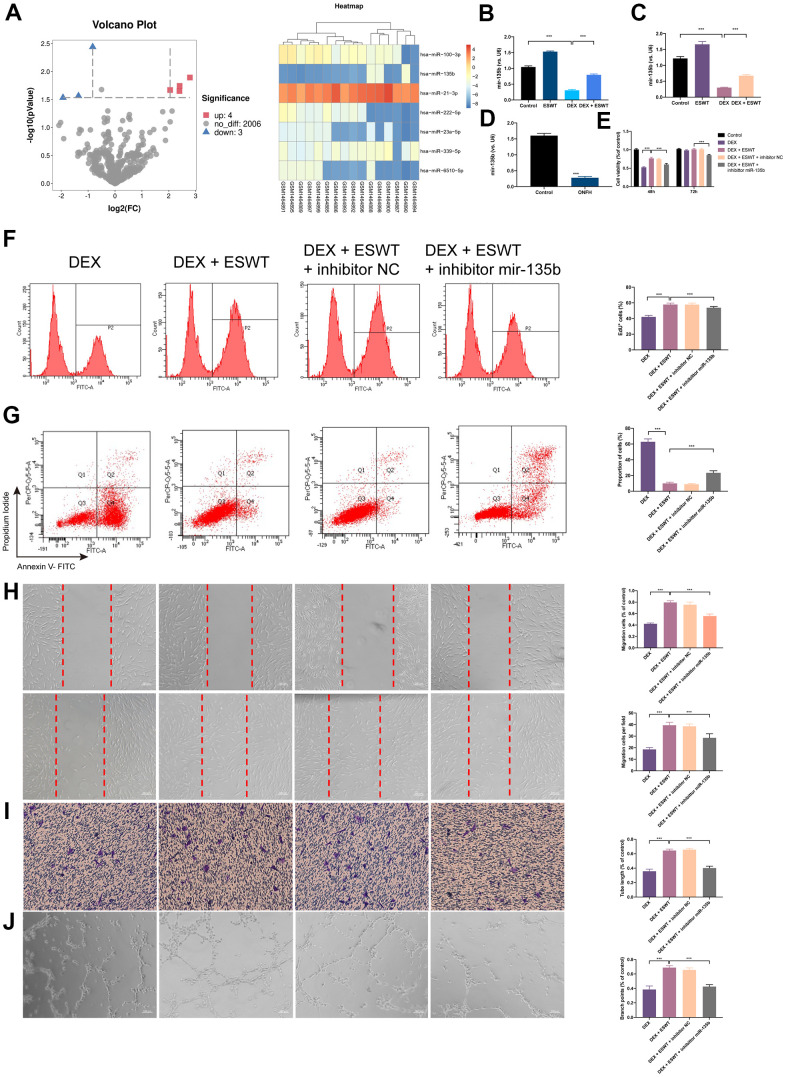
**Effect of miR-135b on endothelial cells treated with GCs.** (**A**) Volcano plot and heat map of potential differentially abundance microRNAs; (**B**) expression of miR-135b in HUVECs; (**C**) expression of miR-135b in BMECs; (**D**) expression of miR-135b in femoral head tissue; (**E**) After transfection of inhibitor mir-135b, ECs were subjected to ESWT with 0.05 mJ/mm^2^, 1000 shots followed by DEX with 180 μM. cell viability examined by CCK-8 analysis in BMECs; (**F**) cell proliferation confirmed by EdU assay in BMECs; After transfection of inhibitor mir-135b, ECs were subjected to ESWT with 0.03 mJ/mm^2^, 1000 shots followed by DEX with 180 μM. (**G**) apoptosis rate of assessed through Annexin V-FITC/PI in BMECs; After transfection of inhibitor mir-135b, ECs were subjected to ESWT with 0.05 mJ/mm^2^, 1000 shots followed by DEX with 180 μM. (**H**) migration ability evaluated by wound healing assay in BMECs; (**I**) migration ability evaluated by Transwell assay in BMECs; (**J**) angiogenesis ability evaluated by tube formation assay in BMECs. n=3 ^**^P < .01, ^***^P < .001.

To identify the differentially expressed miRNAs (DEMs) that were regulated by GCs, miRNAs expression profiles of BMECs treated with GCs (GSE74089) were downloaded from the Gene Expression Omnibus database and analyzed. After normalization of the microarray results, DEMs were identified. As shown in the cluster heat map and volcano plot, a total of seven DEMs were observed in the dataset (Figure 3A). Among the DEMs, four were upregulated, whereas three were downregulated. Considering the most dramatically altered expression levels, we performed PCR assays to validate the miR-135b expression level changes in HUVECs, BMECs, and human necrotic femoral head tissues. The results showed that DEX downregulated miR-135b, whereas ESWT resulted in a significant increasement in the expression level of miR-135b (Figure 3B–3D).

### Knockdown of miR-135b reverses the beneficial effect of ESWT on ECs proliferation

To investigate the role of miR-135b in ESWT-mediated proliferation, ECs were transfected with the miR-135b inhibitor or negative control inhibitor. CCK-8 assays were performed to evaluate the role of miR-135b in proliferation regulation, and the results demonstrated that transfection with the miR-135b inhibitor reversed the proliferation effect of ESWT on both EC types. A similar result was further confirmed using EdU assays (Figure 3E, 3F and Supplementary Figure 3).

### Knockdown of miR-135b reverses the beneficial effect of ESWT on ECs apoptosis

To study the role of miR-135b downregulation in DEX-induced cytotoxicity in ECs, the miR-135b inhibitor was transfected into HUVECs and BMECs. The FC analysis results show a higher apoptosis rate in HUVECs transfected with the miR-135b inhibitor. In addition, a similar result was observed in BMECs. These data indicated that knockdown of miR-135b blocked the beneficial effect of ESWT on ECs apoptosis resistance (Figure 3G and Supplementary Figure 3).

### Knockdown of miR-135b reverses the beneficial effect of ESWT on ECs migration and angiogenesis

We further observed that the knockdown of miR-135b by the inhibitor blocked the beneficial effect of ESWT on ECs migration and angiogenesis. ESWT minimized the effects of the miR-135b inhibitor on HUVEC and BMEC migration using wound-healing assays (Figure 3H, 3I and Supplementary Figure 3). Consistent results were also obtained from transwell assays. In addition, we found that the beneficial effect of ESWT was suppressed by miR-135b knockdown.

### FOXO1 is a direct target of miR-135b in ECs

**Figure 4:**
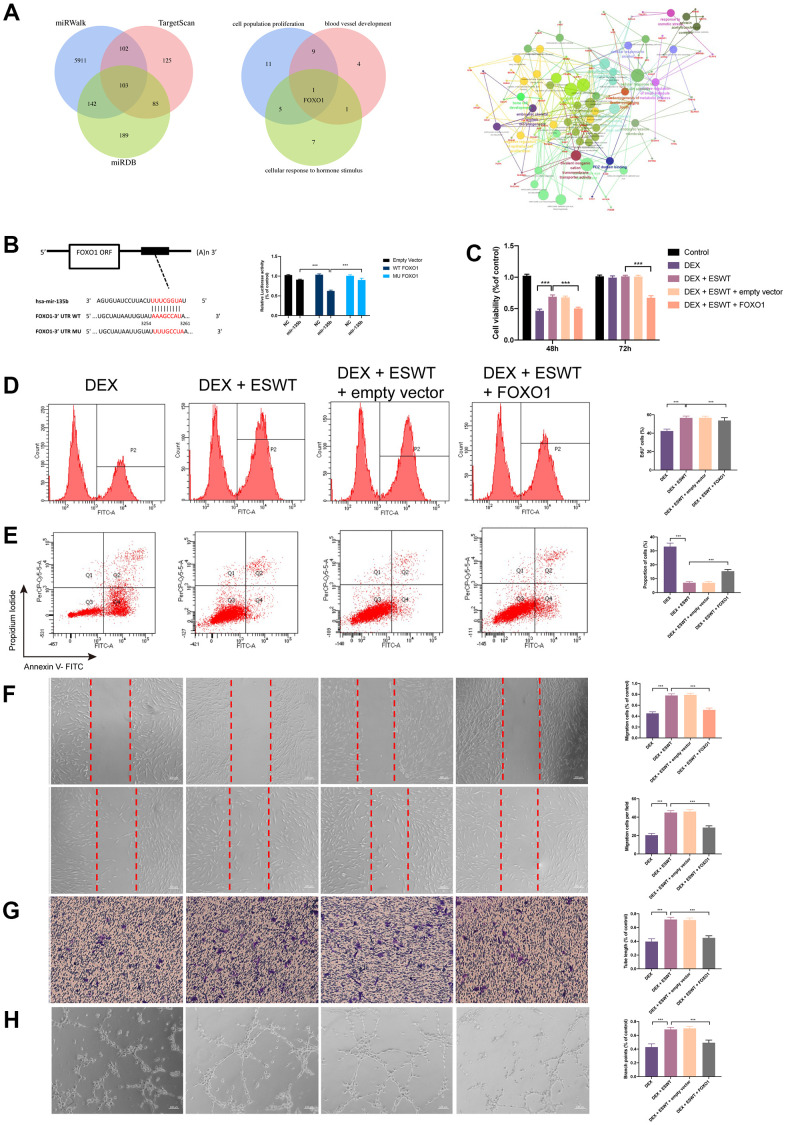
**Effect of FOXO1 on endothelial cells treated with GCs.** (**A**) predicted target gene of miR-135b through miRwalk, TargetScan, and miRDB; GO and KEGG enrichment analysis of potential target genes via ClueGO; bioinformatics analysis revealed FOXO1 as a potential target gene of miR-135b. (**B**) dual luciferase reporter assay verified the targeting relationship between miR-135b and FOXO1; After overexpression of FOXO1, ECs were subjected to ESWT with 0.05 mJ/mm^2^, 1000 shots followed by DEX with 180 μM. (**C**) cell viability examined by CCK-8 analysis in BMECs; (**D**) cell proliferation confirmed by EdU assay in BMECs; After overexpression of FOXO1, ECs were subjected to ESWT with 0.03 mJ/mm^2^, 1000 shots followed by DEX with 180 μM. (**E**) the apoptosis rate assessed through Annexin V-FITC/PI in BMECs; After overexpression of FOXO1, ECs were subjected to ESWT with 0.05 mJ/mm^2^, 1000 shots followed by DEX with 180 μM. (**F**) migration ability evaluated by wound healing assay in BMECs; (**G**) migration ability evaluated by Transwell assay in BMECs; (**H**) angiogenesis ability evaluated by tube formation assay in BMECs. n=3 ^**^P < .01, ^***^P < .001.

To explore the mechanism of miR-135b in ECs proliferation, apoptosis, migration, and angiogenesis, the potential target genes of miR-135b were identified using the TargetScan, miRWalk, and miRDB databases. According to the results, FOXO1, which plays an important role in ECs proliferation, migration, and angiogenesis, maybe a potential target gene of miR-135b (Figure 4A). To test the association between miR-135b and FOXO1, luciferase reporter plasmids of miR-135b and FOXO1 were used. The results demonstrated reduced luciferase reporter activity in the co-transfection of luciferase reporter plasmids containing wild-type FOXO1 and miR-135b mimics in HEK293 cells (Figure 4B). As shown in Figure 4C–4H and Supplementary Figure 4, FOXO1 overexpression significantly inhibited ECs proliferation, apoptosis resistance, migration, and angiogenesis. These results suggest that miR-135b targets and negatively regulates FOXO1 in both HUVECs and BMECs.

### Role of ESWT and miR-135b in the regulation of the FOXO1-cyclin pathway and expression

**Figure 5:**
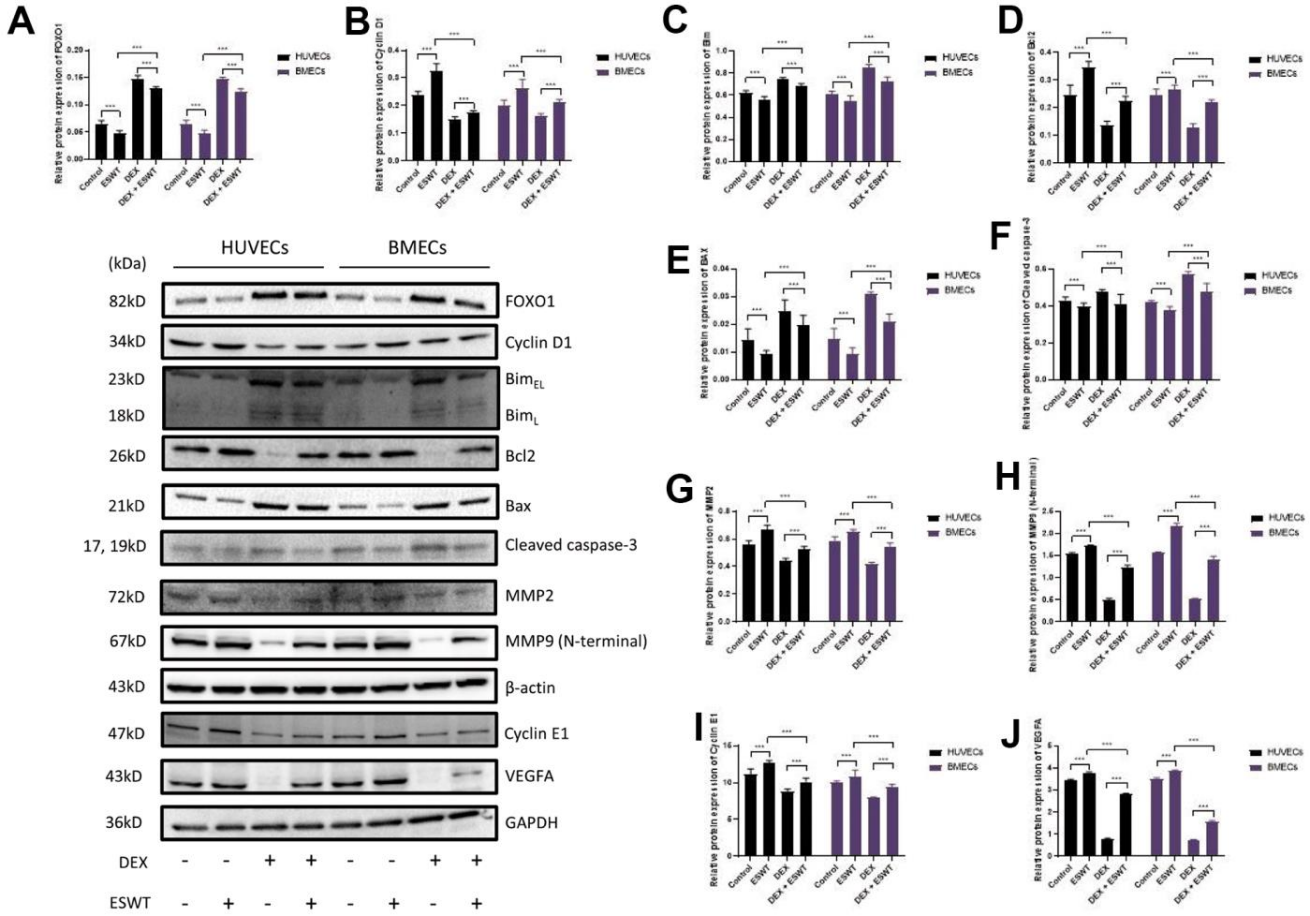
**Role of ESWT in the regulation of FOXO1 and related proteins.** ECs were subjected to ESWT with 0.05 mJ/mm^2^, 1000 shots followed by DEX with 180 μM. (**A**–**J**) Immunoblotting and statistical graph of FOXO1, Cyclin D1, Bim, Bcl2, Bax, cleaved Caspase-3, MMP2, MMP9 (N-terminal), Cyclin E1, and VEGFA. n=3 ^**^P < .01, ^***^P < .001.

**Figure 6:**
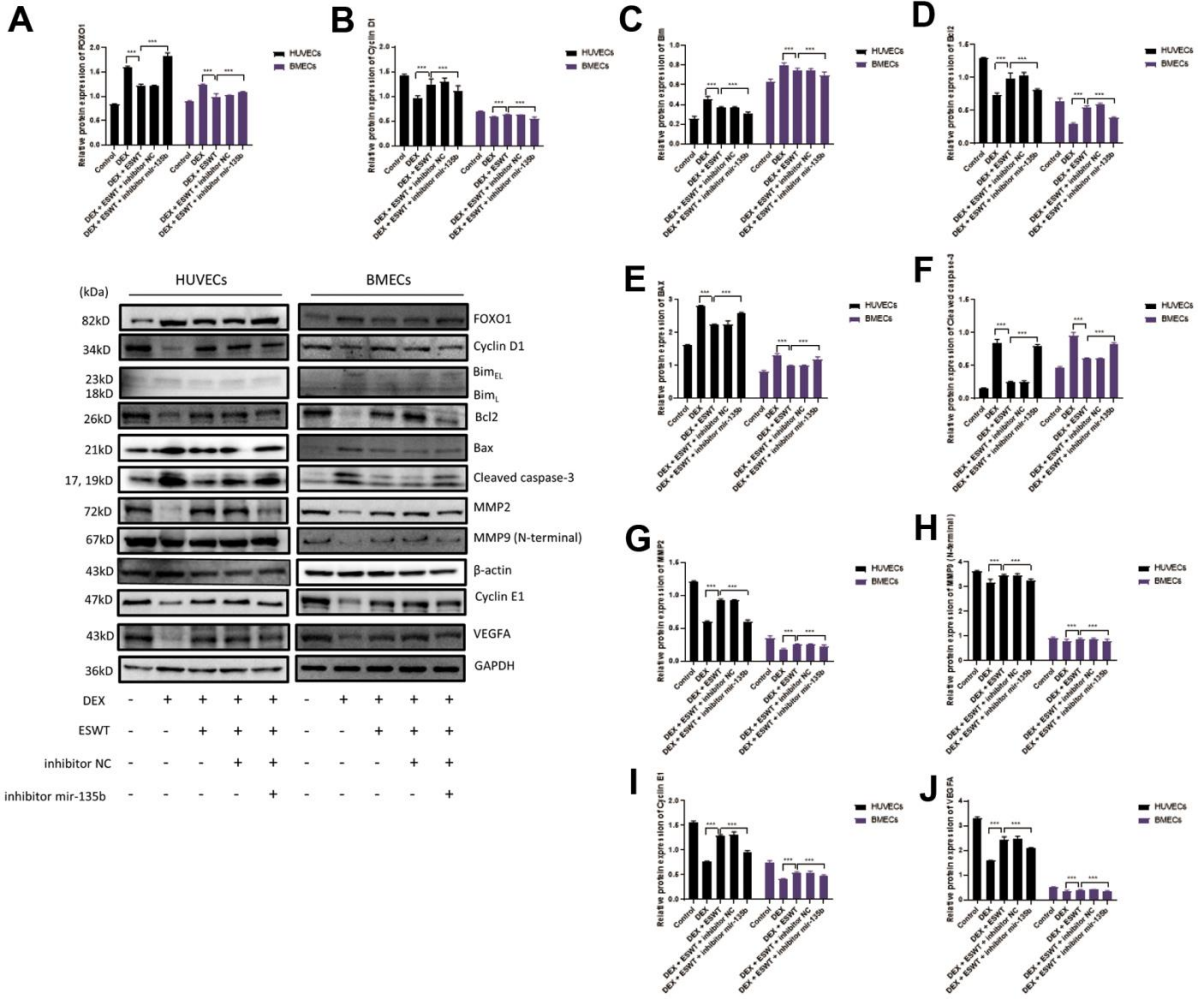
**Role of miR-135b in the regulation of FOXO1 and related proteins.** After transfection of inhibitor mir-135b, ECs were subjected to ESWT with 0.05 mJ/mm^2^, 1000 shots followed by DEX with 180 μM. (**A**–**J**) Immunoblotting and statistical graph of FOXO1, Cyclin D1, Bim, Bcl2, Bax, cleaved Caspase-3, MMP2, MMP9 (N-terminal), Cyclin E1, and VEGFA. n=3 ^**^P < .01, ^***^P < .001.

To further investigate the effects of ESWT on the intracellular pathway and factors related to proliferation, the expression levels of FOXO1, cyclin D1, and cyclin E1 were detected using western blot analysis, which demonstrated that ESWT significantly increased the expression levels of cyclin D1 and cyclin E1 while decreasing the expression level of FOXO1 (Figure 5). Also, we observed that miR-135b knockdown dramatically reversed the beneficial effect of ESWT (Figure 6).

### Role of ESWT and miR-135b in the regulation of the FOXO1-Bim pathway and expression

To further determine whether the roles of ESWT and miR-135b on apoptosis are mediated by the FOXO1-Bim pathway, the expression levels of Bim, Bcl2, Bax, Bad, and cleaved caspase-3 were detected. We found that ESWT significantly decreased the expression levels of pro-apoptotic proteins, such as Bim, Bax, Bad, and cleaved caspase-3, but increased the expression level of Bcl2, an anti-apoptotic protein (Figure 5). Furthermore, miR-135b knockdown blocked the beneficial effect of ESWT (Figure 6).

### Role of ESWT and miR-135b in the regulation of the FOXO1-VEGF pathway and expression

**Figure 7:**
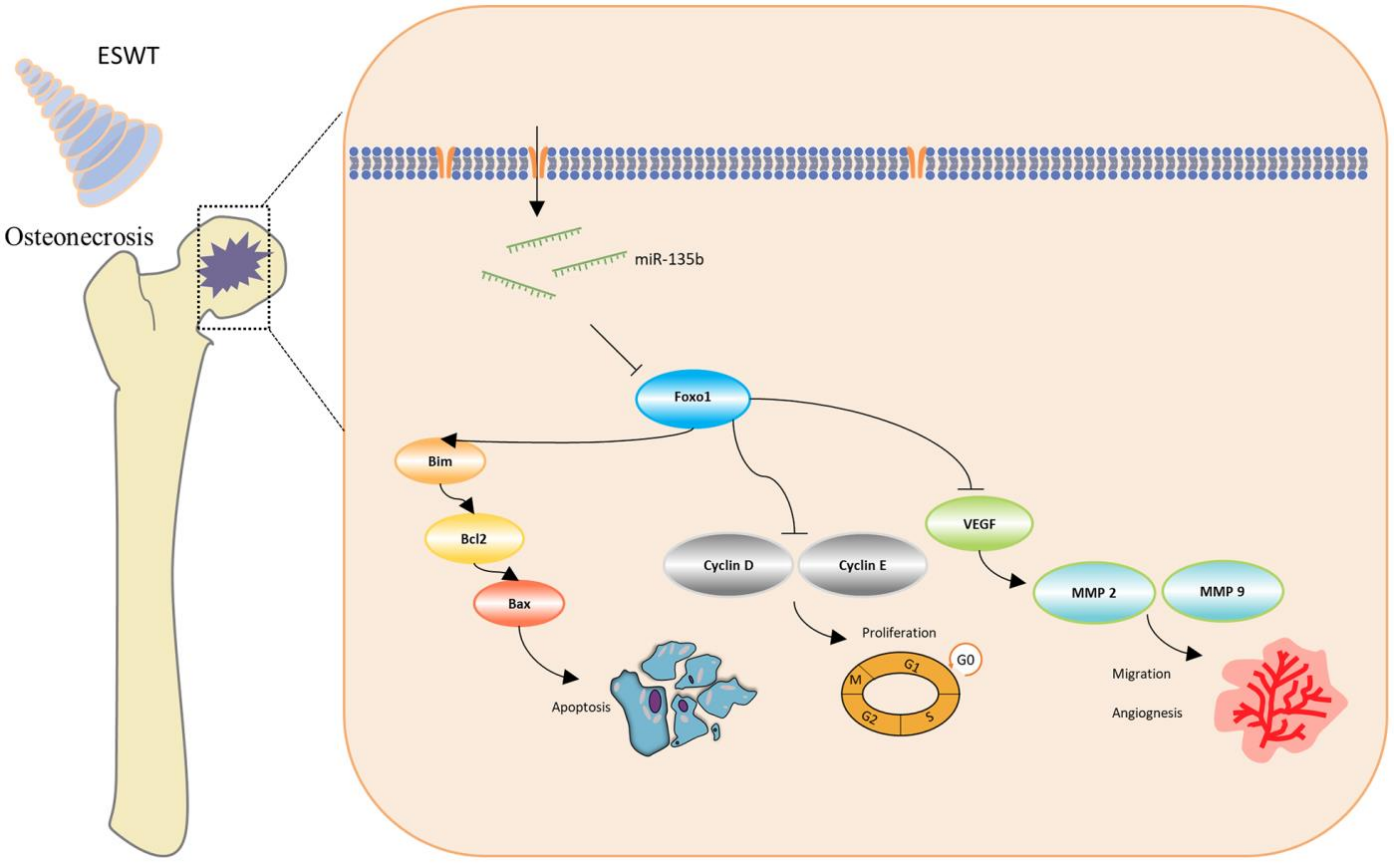
Schematic diagram of the protective effect of ESWT in endothelial cells treated with GCs through miR-135b/FOXO1 pathway.

We further observed that ESWT upregulated migration- and angiogenesis-related proteins, such as MMP2, MMP9, and VEGFA. As expected, ESWT significantly increased the levels of these proteins, which were downregulated owing to DEX (Figure 5). Moreover, miR-135b inhibition markedly suppressed the beneficial effect of ESWT (Figures 6, 7).

## DISCUSSION

In the present study, we demonstrated that ESWT promotes ECs proliferation, migration, and angiogenesis as well as attenuates apoptosis *in vitro* and *in vivo*. *In vitro*, miR-135b downregulation blocked the beneficial effects of ESWT on proliferation, migration, angiogenesis, and anti-apoptosis in both HUVECs and BMECs. Utilizing bioinformatics analysis, FOXO1 was regarded as a potential target gene of miR-135b, and overexpression of FOXO1 was also observed to reverse the positive effect of ESWT. The findings give insights into the role of miRNAs in ESWT for steroid-induced ONFH, clarifying a potential mechanism of the miR-135b/FOXO1 axis.

Previous studies suggested that ECs destruction and dysfunction are strongly related to the pathogenesis of steroid-induced ONFH [12, 13]. Bone development involves bone modeling and remodeling, both of which are coupled with angiogenesis [14–16]. Recently, ESWT has been recognized as an effective therapy for musculoskeletal disorders and ischemic heart disease [17–19]. ESWT could activate local cells, inducing secretion of several cytokines around the diseased tissue, and then exert its effects on the surrounding tissues, including blood vessels and the extracellular environment [20, 21]. In addition, ESWT could increase local metabolism, resulting in tissue repair. In the present study, we firstly investigated the effect of ESWT on vessel volume, ECs proliferation, and apoptosis through *in vivo* study. Then, the optimized parameters of ESWT on ECs were evaluated by *in vitro* studies. We found that ESWT influences ECs in a dose-dependent manner. In a previous study, the results suggested that low-energy ESWT can induce angiogenesis and restore stenotic kidney microcirculation [22]. In another study, low-energy ESWT was also found to be able to ameliorate testicular ischemia-reperfusion injury in rats. This suggests that low energy play a crucial role in the beneficial effects of ESWT, which is consistent with the results of our study.

Now, there is substantial evidence verify that miRNAs play a critical role in the development and progression of steroid-induced ONFH [23, 24]. Therefore, we hypothesize that ESWT may exert beneficial effects through some miRNAs. Hence, we identified DEMs in BMECs treated with GCs compared to healthy controls in our previous study [25]. Through bioinformatics analysis, we found that miR-135b may be the potential mediator, which has been known to negatively regulate the expression of FOXO1 in both ECs and osteoblasts [26, 27]. Consistently, we also found that miR-135b expression was downregulated in steroid-induced ONFH rats, HUVECs, and BMECs, accompanied by conversely-upregulated FOXO1. In addition, when exposed to ESWT, GCs failed to repress miR-135b expression.

FOXO1 has been reported to exert broader suppressive activity in regulating ECs proliferation, migration, and angiogenesis while promoting apoptosis [28, 29]. Using bioinformatics analysis, our results revealed that FOXO1 may serve as a target gene of miR-135b. A recent study indicated that FOXO1 represses cyclin D1 expression, thereby promoting cell cycle arrest [30]. Our study also suggested that ESWT-induced upregulation of miR-135b and downregulation of FOXO1 may contribute to increased cyclin D1 and E1 levels. Conversely, the beneficial effect of ESWT is abolished when miR-135b is inhibited and FOXO1 is upregulated.

Notably, activation of FOXO1 initiates apoptosis by targeting the downstream molecules Bim, Bcl2, and Bax [31]. Bim plays a critical role in the pro-apoptotic process by interacting with anti-apoptotic proteins. FOXO1 could directly activate Bim gene expression to promote apoptosis [32]. In the present study, DEX was observed to induce Bim production, thereby leading to apoptosis. In addition, ESWT could prevent ECs from apoptosis both *in vitro* and *in vivo*.

Intriguingly, VEGF, a potent angiogenic factor, plays a vital role in ECs proliferation, migration, and angiogenesis [33]. The MMP superfamily consists of metalloproteinases that degrade the extracellular matrix, a fundamental process before cell migration, invasion, and angiogenesis [34, 35]. In a previous study investigating the effects of the VEGF- related pathway in ESWT, the results suggest that ESWT significantly enhances diabetic wound healing [36]. The mechanism mainly involves increasing neovascularization and tissue regeneration via VEGF and MAPK mediated pathway. Importantly, FOXO1, one of the most widely studied members of the FOXO family, is an essential regulator of endothelial growth. Previous studies have shown that endothelial-restricted deletion of FOXO1 induces a profound increase in ECs proliferation and vessel development [37]. In our study, the results suggested that FOXO1 was a target of miR-135b and altered the expression levels of VEGFA, MMP2, and MMP9, thereby regulating migration and angiogenesis in both HUVECs and BMECs.

Our study presented some limitations. Firstly, many signal pathways regulate proliferation, apoptosis, migration, and angiogenesis; however, our present study only investigated main signal pathways according to previous studies. Further studies should investigate more. Secondly, the dose-timing effects of miR-135b on rats were not assessed. Further studies are needed to elucidate that.

In conclusion, our study indicates that ESWT relieves endothelial injury and dysfunction in steroid-induced ONFH via miR-135b targeting of FOXO1.

## Supplementary Material

Supplementary Figures

Supplementary Table 1
