# Supplementary material for: Extracorporeal shockwave relieves endothelial injury and dysfunction in steroid-induced osteonecrosis of the femoral head via miR-135b targeting FOXO1: in vitro and in vivo studies
Source: Aging (Albany NY). 2022 Jan 7;14(1):410–29. doi: 10.18632/aging.203816 (PMC8791199; doi:10.18632/aging.203816)
Supplement: Supplementary Figures [file aging-14-203816-s001.pdf]

# SUPPLEMENTARY FIGURES

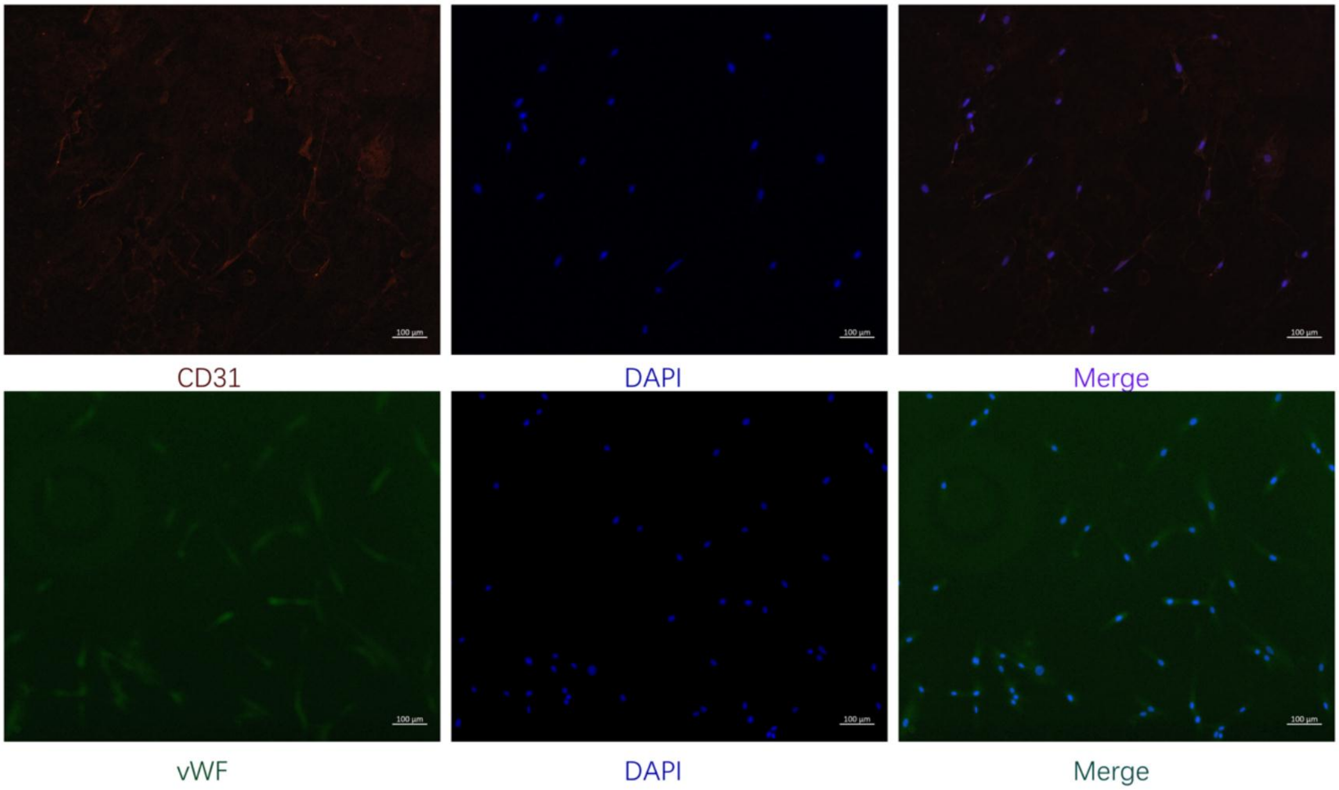


**Supplementary Figure 1. Identification of BMECs.** The representative CD31-positive and vWF-positive cells were identified as BMECs.

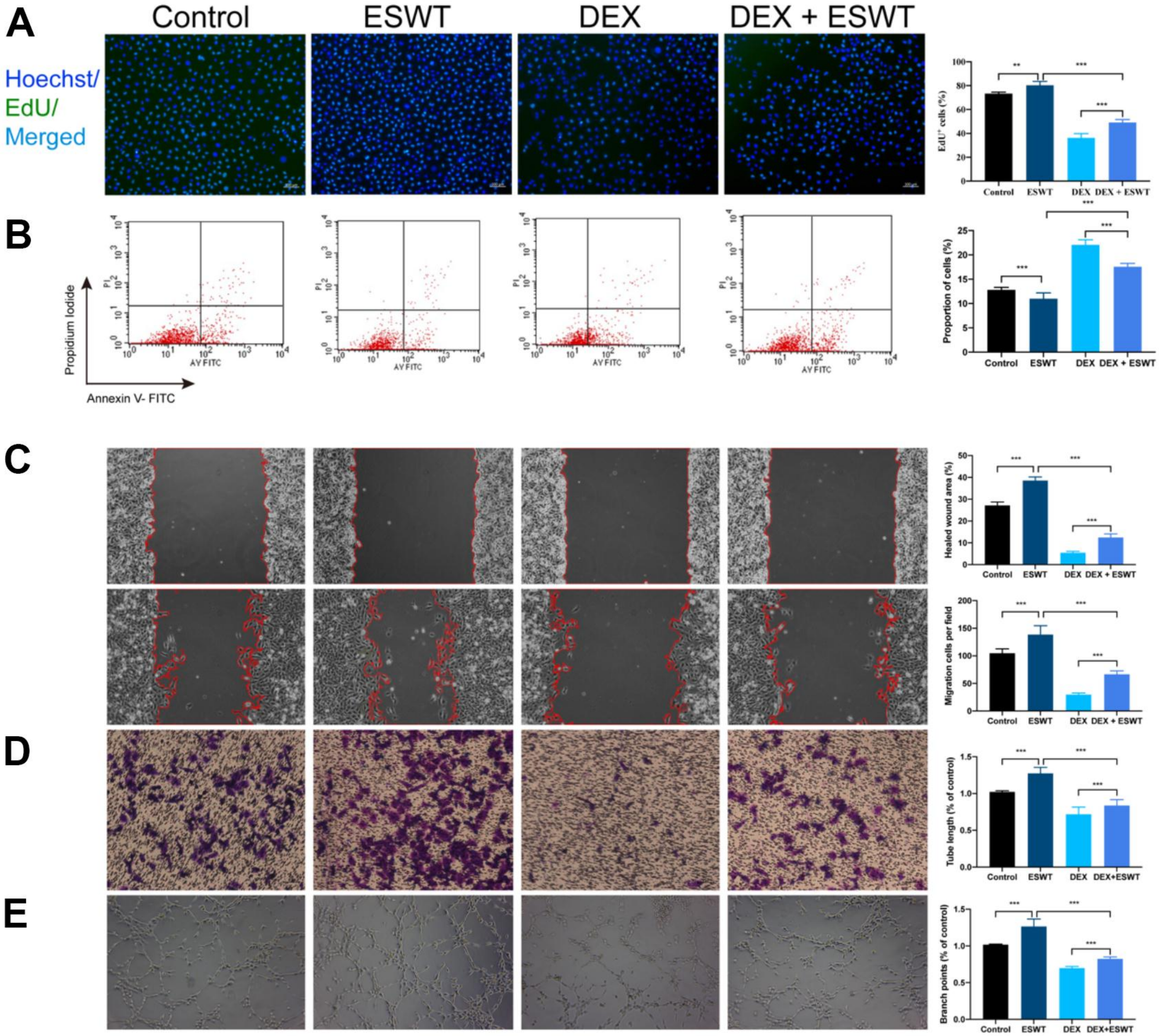


**Supplementary Figure 2. Effect of ESWT on HUVECs treated with GCs.** ECs were subjected to ESWT with 0.05 mJ/mm$^2$, 1000 shots followed by DEX with 180 µM. (**A**) cell proliferation confirmed by EdU assay; (**B**) apoptosis rate of assessed through Annexin V-FITC/PI; (**C**) migration ability evaluated by wound healing assay; (**D**) migration ability evaluated by Transwell assay; (**E**) angiogenesis ability evaluated by tube formation assay. n=3 $^{**}$P < .01, $^{***}$P < .001.

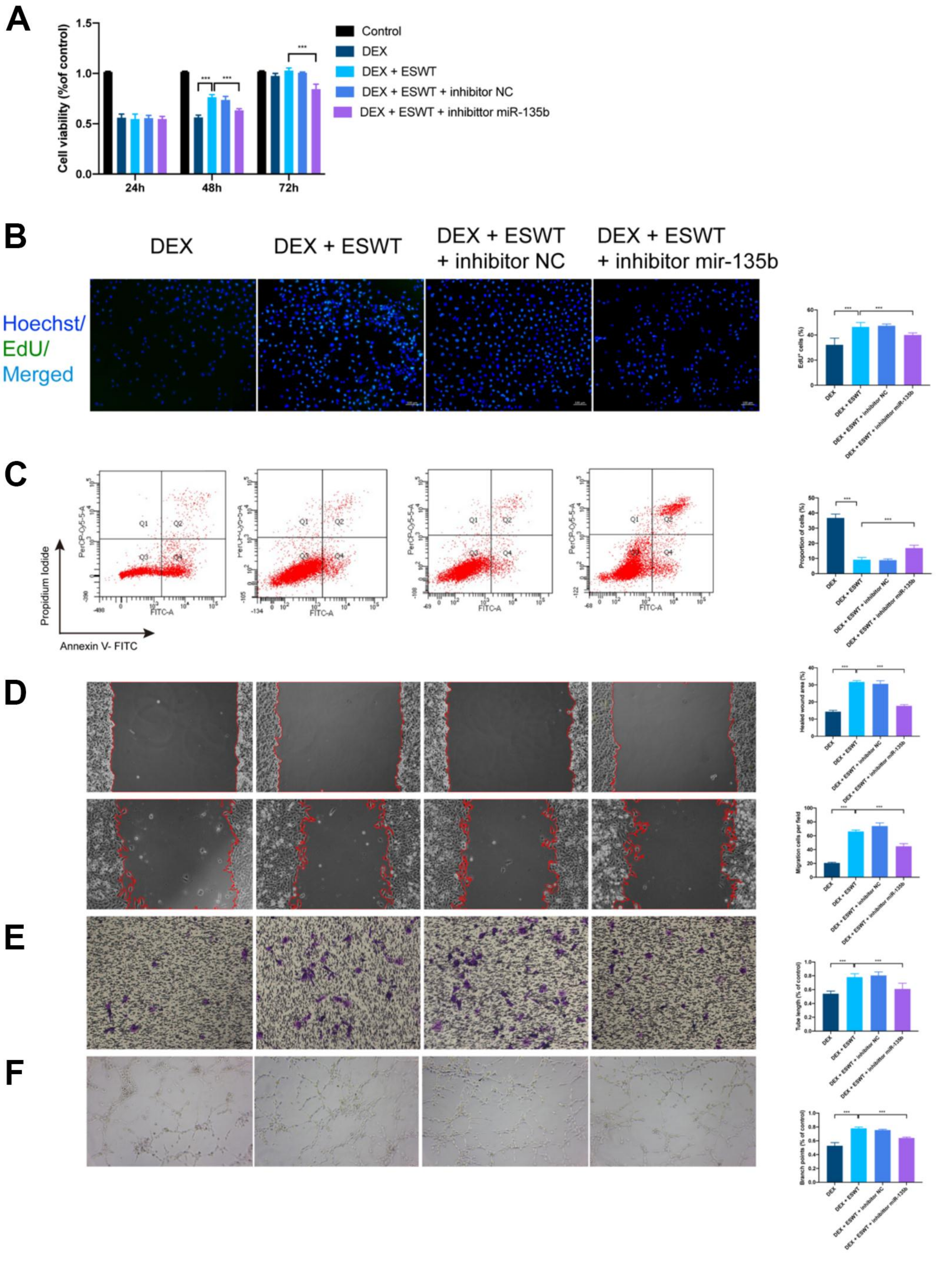


**Supplementary Figure 3. Effect of miR-135b on HUVECs treated with GCs.** After transfection of inhibitor mir-135b, ECs were subjected to ESWT with 0.05 mJ/mm$^2$, 1000 shots followed by DEX with 180 μM. (**A**) cell viability examined by CCK-8 analysis; (**B**) cell proliferation confirmed by EdU assay; (**C**) apoptosis rate of assessed through Annexin V-FITC/PI; (**D**) migration ability evaluated by wound healing assay; (**E**) migration ability evaluated by Transwell assay; (**F**) angiogenesis ability evaluated by tube formation assay. n=3 $^{**}$P < .01, $^{***}$P < .001.

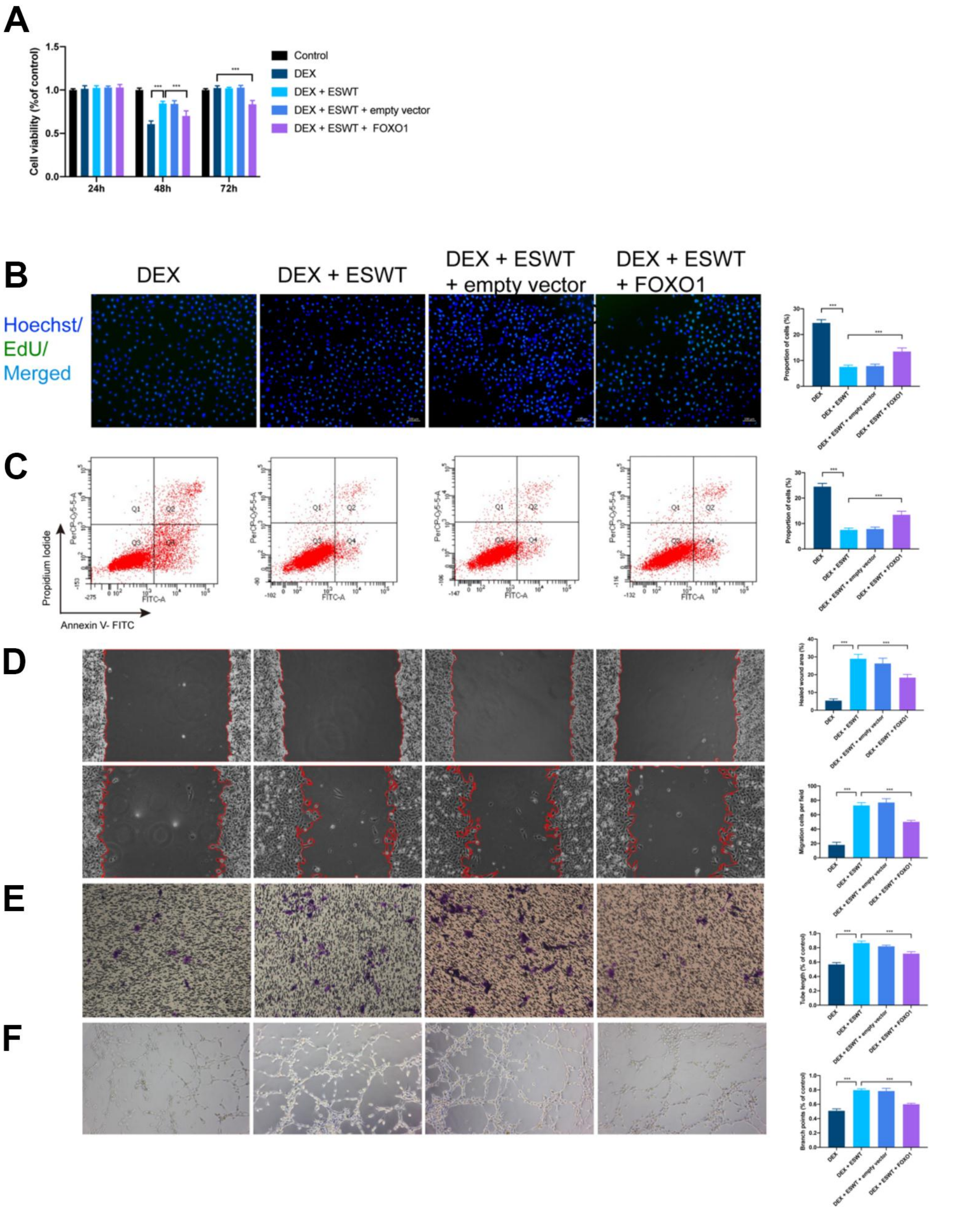


**Supplementary Figure 4. Effect of FOXO1 on HUVECs treated with GCs.** After overexpression of FOXO1, ECs were subjected to ESWT with 0.05 mJ/mm$^2$, 1000 shots followed by DEX with 180 μM. (**A**) cell viability examined by CCK-8 analysis; (**B**) cell proliferation confirmed by EdU assay; (**C**) apoptosis rate of assessed through Annexin V-FITC/PI; (**D**) migration ability evaluated by wound healing assay; (**E**) migration ability evaluated by Transwell assay; (**F**) angiogenesis ability evaluated by tube formation assay. n=3 $^{**}P < .01$, $^{***}P < .001$.
