# Supplementary material for: Extracorporeal shockwave relieves endothelial injury and dysfunction in steroid-induced osteonecrosis of the femoral head via miR-135b targeting FOXO1: in vitro and in vivo studies
Source: Aging (Albany NY). 2022 Jan 7;14(1):410–29. doi: 10.18632/aging.203816 (PMC8791199; doi:10.18632/aging.203816)
Supplement: Supplementary Table 1 [file aging-14-203816-s002.pdf]

SUPPLEMENTARY TABLE

Supplementary Table 1. Clinical details of patients for isolation of bone microvascular endothelial cells.

Patients	Disease	Age (years)	Gender	Height (cm)	Weight (kg)	Garden grade
P1	FNF	67	M	172	64	IV
P2	FNF	68	M	171	66	IV
P3	FNF	68	M	170	70	IV
P4	FNF	66	M	175	62	IV
